# Severe Neurological Presentation in Siblings With *COQ5* ‐Related Primary Coenzyme Q10 Deficiency: Expanding Clinical and Molecular Spectrum

**DOI:** 10.1002/jmd2.70038

**Published:** 2025-11-05

**Authors:** Parith Wongkittichote, Rachel M. Guerra, Daniel J. Wegner, Samantha Toy, Jacqueline A. Hauer, David J. Pagliarini, Jorge L. Granadillo

**Affiliations:** ^1^ Division of Genetics and Genomic Medicine, Department of Pediatrics Washington University School of Medicine in St. Louis St. Louis Missouri USA; ^2^ Department of Pediatrics Faculty of Medicine Ramathibodi Hospital, Mahidol University Bangkok Thailand; ^3^ Department of Cell Biology and Physiology Washington University School of Medicine in St. Louis St. Louis Missouri USA; ^4^ Edward Mallinckrodt Department of Pediatrics Washington University in St. Louis School of Medicine and St. Louis Children's Hospital St. Louis Missouri USA; ^5^ Department of Pediatrics Mercy Children's Hospital Springfield Missouri USA; ^6^ Department of Biochemistry and Molecular Biophysics and Department of Genetics Washington University School of Medicine St. Louis Missouri USA; ^7^ Department of Genetics Washington University School of Medicine St. Louis Missouri USA; ^8^ Howard Hughes Medical Institute, Washington University School of Medicine St. Louis Missouri USA

## Abstract

Coenzyme Q_10_ (CoQ_10_) is a coenzyme and antioxidant involved in multiple bioenergetic and biosynthetic processes, particularly within mitochondria. The biosynthesis of CoQ_10_ is a tightly regulated process that involves multiple enzymes, including the methyltransferase COQ5. Genetic defects in *COQ5* have recently been associated with autosomal recessive *COQ5*‐related primary CoQ_10_ deficiency. The clinical manifestations of seven individuals previously reported were primarily neurological and ophthalmological. Here, we report two siblings with profound developmental delay and brain imaging consistent with multistage strokes. Clinical exome sequencing revealed compound heterozygous variants in *COQ5*, including one frameshift deletion and one missense variant. Our functional complementation studies demonstrate that a *Saccharomyces cerevisiae COQ5* ortholog harboring the corresponding missense variant fails to fully rescue *coq5*∆ CoQ_6_ production, leading to the accumulation of CoQ biosynthetic intermediates. After the diagnosis, CoQ_10_ supplementation was started on the proband, leading to subjective clinical improvement. We describe new cases of *COQ5*‐related primary CoQ_10_ deficiency and expand the phenotypic and molecular spectrum of the disease. We also establish a yeast system to evaluate the effects of the variants in *COQ5* and support the use of CoQ_10_ supplementation for patients with *COQ5*‐related primary CoQ_10_ deficiency.

**Summary**

- This study expands the clinical spectrum of *COQ5*‐related primary CoQ_10_ deficiency to include severe neurological abnormalities with multi‐stage strokes.
- It also validates a yeast‐based functional assay using targeted lipidomics, providing a valuable tool for characterizing human *COQ5* variants.

## 1 Introduction

Coenzyme Q_10_ (CoQ_10_), composed of a benzoquinone head group and a long polyisoprenyl tail, is a coenzyme and antioxidant used in multiple cellular processes, including the mitochondrial electron transport chain, pyrimidine biosynthesis, ferroptosis defense, and the maintenance of cellular redox balance [1, 2, 3]. The biosynthesis of CoQ_10_ is a tightly regulated multistep process involving multiple enzymes [2]. Defects in CoQ_10_ biosynthesis have been associated with a rare group of heterogeneous disorders, namely primary CoQ_10_ deficiency [4, 5]. They share overlapping features including developmental delay, encephalopathy, epilepsy, ataxia, and myopathy. Additional presentations include steroid‐resistant nephrotic syndrome, hypertrophic cardiomyopathy, optic atrophy, and retinopathy [6, 7]. The patients may exhibit abnormal biochemical analysis including reduced CoQ_10_ levels in muscle, fibroblast, or leukocytes, or abnormal mitochondrial transport enzymes activity [6].

To date, at least 13 enzymes are known to be associated with CoQ_10_ metabolism [2, 4]. Briefly, 4‐hydroxybenzoic acid (4‐HB), generated from tyrosine, is linked to polyisoprenyl pyrophosphate to form 3‐hexaprenyl‐4‐hydroxy benzoic acid (PPHB), which is further modified by multiple enzymes, including an S‐adenosylmethionine‐dependent methyltransferase encoded by *COQ5* (MIM# 616359), located on chromosome 12q24.31. In Baker's yeast ( *Saccharomyces cerevisiae* ), the knock‐out of the human orthologue *COQ5* (*coq5*Δ) displayed an oxidative growth defect and accumulation of its substrate, demethyl‐demethoxy‐Q_6_ (DDMQ_6_) [8].

*COQ5*‐related primary CoQ_10_ deficiency (*COQ5*‐PCD) is an extremely rare autosomal recessive disorder that was initially described in three siblings born to non‐consanguineous parents of Iraqi‐Jewish descent who presented with cerebellar ataxia, epilepsy, developmental, and cognitive delay [9]. Biochemical studies showed reduced leukocyte and muscle CoQ_10_ levels. Genetic testing revealed a homozygous exon duplication causing an abnormally long 3′ untranslated region (3′UTR). Four additional cases have been reported with a combination of missense, splicing, and truncating variants [10, 11, 12]. These findings indicate that loss of function is a presumptive molecular mechanism. However, these studies did not assess the effect of these variants on CoQ_10_ metabolism. Moreover, the lack of a functional assay made assessment of missense variants challenging.

Here we identified two novel variants in *COQ5* in two siblings with profound developmental delay, epilepsy, hypotonia, and stroke‐like episodes via clinical exome sequencing. We also employ complementation studies in *S. cerevisiae* lacking a Coq5 ortholog (*coq5*∆) to analyze the biochemical effects of this missense variant. We reveal that the yeast Coq5 ortholog harboring the corresponding mutation fails to rescue CoQ_6_ levels (the *S. cerevisiae* equivalent of human CoQ_10_) and results in the accumulation of CoQ_6_ intermediates, thereby supporting the likely pathogenicity of this variant.

## 2 Materials and Methods

### 2.1 Case Description

#### 2.1.1 Patient 1

**FIGURE 1:**
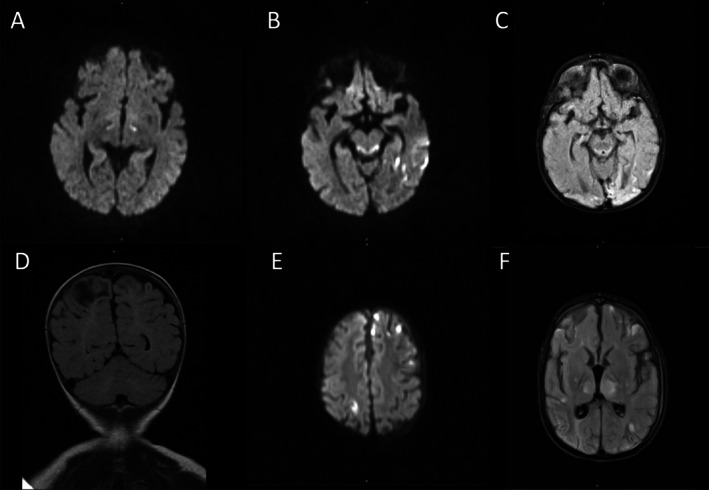
Brain imaging of the probands. (A–C) Brain MRI of Patient 1: (A) Brain MRI at the age of 21 months of age demonstrated reduced diffusivity in bilateral thalami on diffusion weighted imaging (B) At the age of 25 months, brain MRI showed reduced diffusivity in bilateral dorsal midbrain and left dorsal cerebral cortices on diffusion weighted imaging. (C) At 33 months of age, brain MRI showed gliosis in brainstem and left dorsal cerebral cortices as well as evolving ischemic injury in right dorsal cerebral cortices on T2/FLAIR imaging. (D–F) Brain MRI of Patient 2: (D) Brain MRI at 3 months of age demonstrated encephalomalacia in right greater than left parietal cortices on T2/FLAIR imaging. (E–F) Brain MRI at 3 years of age showed multifocal distribution of decreased diffusivity on diffusion weighted imaging (E) and hyperintensity on T2/FLAIR imaging (F).

The proband was a 4‐year‐old white female who was born to a 26‐year‐old mother. Pregnancy was uncomplicated. She was born at 39 weeks 1 day of estimated gestational age (EGA) via planned cesarean section. Delivery and neonatal courses were uneventful. Her birth weight was at the 25^th^ percentile, birth length was at the 59^th^ percentile, and occipitofrontal circumference (OFC) was at the 4^th^ percentile. She was noted to have developmental delay at 6 months of age. Family history was notable for three healthy siblings and a brother who also had developmental delay (Patient 2). Brain magnetic resonance imaging (MRI) at 21 months of age showed reduced diffusivity within the bilateral ventral thalami and cerebral peduncles with diffuse nonspecific white matter volume loss (Figure 1A). Follow‐up brain MRI at 25 months of age showed worsening signal abnormality within the dorsal midbrain and medial thalami and new abnormality involving the left PCA territory suggestive of subacute infarct (Figure 1B). Head and neck magnetic resonance angiography (MRA) did not detect significant stenosis or occlusion. Brain MRI at 33 months showed gliosis of previously demonstrated areas of ischemic injury in thalami, brainstem, and left dorsal cerebral cortices, and new areas of late subacute to chronic ischemic injury in right posterior temporal and occipital cortices (Figure 1C).

She developed new onset seizure at 26 months of age. Electroencephalography (EEG) revealed abnormal spike and wave complexes predominantly in the left temporooccipital head region and synchronously in the right temporoparietooccipital head region. Later EEG at 60 months of age showed multifocal epileptiform discharges and generalized seizures. Repeated brain MRI showed stable abnormal signal intensity in bilateral medial thalami and dorsal midbrain and mild decrease in the edema associated with the suspected subacute infarct. She also had feeding difficulties and underwent gastrostomy tube placement at the age of 20 months due to failure to gain appropriate weight.

Upon evaluation at 27 months, she was able to roll over but could not sit or stand independently. She was able to reach out and transfer objects but could not use a pincer grasp. She could smile and coo but was nonverbal. Her weight and OFC were both below the 1^st^ percentile, with *Z*‐scores of −6.07 and −2.89, respectively. Her height was at the 1st percentile. Physical examination was notable for microcephaly and bilateral epicanthal folds but otherwise nondysmorphic. Neurologic examination was notable for sluggish pupillary reaction, axial hypotonia with spasticity of lower extremities, and generalized hyperreflexia. Ophthalmologic examination at the age of 4 years revealed ptosis, pale optic discs concerning for optic atrophy, and abnormal electroretinography consistent with retinopathy.

She underwent extensive metabolic evaluation including unremarkable total and free carnitine, acylcarnitine profile, serum amino acids, homocysteine, urine organic acids, creatine kinase, biotinidase, peroxisomal profile, and ammonia. Lactate was elevated with a maximum of 4.4 mmol/L (reference range 0.6–2.0 mmol/L) with a normal lactate: pyruvate ratio. Mitochondrial electron transport enzymes analysis in fibroblasts was normal. CoQ_10_ level in leukocytes was reduced to 38 pmol/mg protein (normal range 66–183 pmol/mg). Chromosomal microarray was normal.

At 37 months of age, supplementation with 15 mg/kg/day of CoQ10 was initiated. Within 2 months, her parents noted subjective clinical improvement in social interaction. Brain MRI at 40 and 45 months showed no new infarctions. From 42 months of age, CoQ10 supplementation was inconsistently given and eventually stopped. Brain MRI at 60 months showed new infarctions in the right posterior temporal occipital cortices.

#### 2.1.2 Patient 2

Patient 2 is an elder brother of Patient 1. He was 7 years of age at the time of evaluation. He first presented with seizures at 3 months of age. Brain MRI at that time showed evolving encephalomalacia in bilateral parietal lobes (Figure 1D). EEG showed epileptiform discharges in the right central head region as well as clinicoelectrographic and subclinical seizures with onset in the same region. At 3 years of age, he presented with recurrent lactic acidosis and strokes in the setting of dehydration. Brain MRI at that time showed multiple strokes of varying ages, from acute/subacute to chronic (Figure 1E,F). It also showed hyperintensities in the bilateral thalami, as well as the brainstem, without corresponding diffusionopathy. He had a history of cerebral palsy, epilepsy, and profound intellectual disability. He was gastrostomy tube dependent, non‐verbal, and did not walk.

Unfortunately, he died at home at age 7 years due to unknown causes. Regarding laboratory evaluations, his lactate was normal. Leukocyte CoQ_10_ level was reduced to 39 pmol/mg protein (normal range 66–183 pmol/mg).

Clinical quad exome sequencing of Patient 1, Patient 2, and their parents revealed compound heterozygous variants in *COQ5* (NM_032314.4) in both individuals: paternally inherited 2‐bp deletion, designated as c.177_178del (p.Ser60Glyfs13) and a maternally inherited missense variant, namely c.353G>A (p.Gly118Asp).

### 2.2 Next Generation Sequencing

Clinical exome sequencing was performed by GeneDx (Gaithersburg, MD). Bioinformatic analysis was performed based on the company's pipeline. *In silico* analysis of the effect of variants was performed by various bioinformatic tools including Splice AI [13], Combined Annotation Dependent Depletion (CADD) [14], Rare exome variant ensemble learner (Revel) [15], VARITY [16], Sorting Intolerant From Tolerant (SIFT) [17], MutationTaster2 (MT) [18] and Functional Analysis through Hidden Markov Models (FATHMM) [19]. Variant classification was performed according to the American College of Medical Genetics and Genomics (ACMG) recommendation [20].

### 2.3 Yeast Strains and Cultures

The *S. cerevisiae* haploid strain W303 (Matα; leu2‐3; trp1‐1; can1‐100; ura3‐1; ade2‐1; his3‐11) was used. The *coq5*Δ strain with deletion of yeast *COQ5* was generated using PCR‐based homologous recombination, replacing the open reading frame of the targeting gene with the NatMX6 cassette [21]. The p416 plasmid with *S. cerevisiae* Coq5‐Flag expressed under a GPD promoter [22] was used as a template to generate the p.Gly123Asp point mutation via site‐directed mutagenesis. W303 WT and *coq5*Δ strains were transformed with p416 empty vector, p416‐GPD‐Coq5‐Flag WT, or p416‐GPD‐Coq5‐Flag G123D, using the LiAc/SS carrier DNA/PEG method [23] and plated on synthetic deficient (SD) Ura^−^ media with 2% glucose (US Biological). For lipid extraction and immunoblotting, 5 mL cultures of SD Ura, 2% glucose were inoculated with individual colonies and incubated (30°C, 230 r.p.m., 14–16 h). Cell density was measured as an optical density at 600 nm (OD_600_) and converted to cells mL^−1^ (1 OD = 1 × 10^7^ cells mL^−1^). 1 × 10^8^ cells were collected, pelleted, and snap‐frozen in LN_2_, and stored at −80°C.

### 2.4 Lipid Extraction and LC–MS Lipidomics

For lipid extractions, frozen cell pellets were thawed on ice, then 150 mM KCl (50 μL) was added to each sample, followed by ice‐cold methanol (600 μL; with 1 μM CoQ_8_ as internal standard). Glass beads (100 μL; 0.5 mm; BioSpec) were added, and the samples were vortexed (10 min, 4°C) to lyse the cells. Ice‐cold petroleum ether (400 μL) was added to extract the lipids, and the samples were vortexed again (3 min, 4°C). Samples were centrifuged (1000 × g, 3 min, RT) and the top petroleum ether layer was collected in a new tube. The petroleum ether extraction was repeated a second time, with the petroleum ether layer from the second extraction combined with that from the first. The extracted lipids were dried under argon before being resuspended in 2‐propanol (50 μL) and transferred to an amber glass vial (Sigma; QSertVial, 12 × 32 mm, 0.3 mL).

LC–MS analysis was performed using a Thermo Vanquish Horizon UHPLC system coupled to a Thermo Exploris 240 Orbitrap mass spectrometer. For LC separation, a Vanquish binary pump system (Thermo Fisher Scientific) was used with a Waters Acquity CSH C18 column (100 × 2.1 mm, 1.7 μm particle size) held at 35°C under a 300 μL/min flow rate. Mobile phase A consisted of 5 mM ammonium acetate in acetonitrile:H_2_O (70:30, v/v) with 125 μL/L acetic acid. Mobile phase B consisted of 5 mM ammonium acetate in isopropanol: acetonitrile (90:10, v/v) with the same additive. For each sample run, mobile phase B was initially held at 2% for 2 min and then increased to 30% over 3 min. Mobile phase B was further increased to 50% over 1 min and 85% over 14 min and then raised to 99% over 1 min and held for 4 min. The column was re‐equilibrated for 5 min at 2% B before the next injection. Five microliters of the sample were injected by a Vanquish Split Sampler HT autosampler (Thermo Fisher Scientific), while the autosampler temperature was kept at 4°C. The samples were ionized by a heated ESI source kept at a vaporizer temperature of 350°C. Sheath gas was set to 50 units, auxiliary gas to 8 units, sweep gas to 1 unit, and the spray voltage was set to 3500 V for positive mode and 2500 V for negative mode. The inlet ion transfer tube temperature was kept at 325°C with 70% RF lens. For targeted analysis, the MS was operated in parallel reaction monitoring mode with polarity switching acquiring scheduled, targeted scans to CoQ_6_ (m/z 591.4408), CoQ_8_ (m/z 727.566), and CoQ intermediates: DDMQ_6_ (m/z 547.4146) and PPHB_6_ (m/z 545.4). MS acquisition parameters include a resolution of 15,000, stepped HCD collision energy (25%, 30%, 40% for positive mode and 20%, 40%, 60% for negative mode), and 3 s dynamic exclusion. Automatic gain control targets were set to standard mode. The resulting CoQ intermediate data were processed using TraceFinder 5.1 (Thermo Fisher Scientific).

### 2.5 Western Blotting

Frozen yeast pellets were lysed in 150 μL of lysis buffer (1 M NaOH, 1 M BME) for 10 min with periodic vortexing. Protein was precipitated with 150 μL of 50% TCA and washed with 1 mL of acetone. The protein pellet was resuspended in 50 μL of 0.1 M NaOH and 25 μL of 3X LDS sample buffer. Proteins were separated (200 V, 35 min), transferred to a PVDF membrane (Sigma), and blocked with 5% non‐fat dry milk (1 h, RT.). Membranes were then probed with primary anti‐FLAG (Sigma, F1804, 1:1000) or anti‐actin (Sigma, 8224, 1:1000) antibodies (1 h, RT). Membranes were washed three times with TBST and then probed with HRP‐linked anti‐mouse IgG (Cell Signaling, #7076, 1:5000) secondary antibody (1 h, RT). Membranes were washed three times with TBST and developed using SignalFire ECL reagent (Cell Signaling). Developed membranes were imaged on a ChemiDoc system (Bio‐Rad).

## 3 Results

### 3.1 Molecular Analysis of *COQ5* Variants

Compound heterozygous variants in *COQ5* (NM_032314.4) were detected in both patients: c.177_178del (p.Ser60Glyfs13) and c.353G>A (p.Gly118Asp). The 2‐bp deletion variant likely causes a frameshift, resulting in either nonsense mediated decay or protein truncation leading to the loss‐of‐function of the protein. *In silico* prediction of p.Gly118Asp supported a deleterious effect on enzyme function (Table 1). The base position c.353G is a splice acceptor site; however, *in silico* analysis for abnormal splicing was inconclusive [13]. We then performed an RNA study which revealed that the variant p.Gly118Asp does not affect splicing (Figure S1). None of the unaffected siblings are compound heterozygous for these two variants.

**TABLE 1:** *In silico* prediction and ACMG classification of missense variant.

	c.353G>A (p.Gly118Asp)
Splice AI	Splice‐Altering/moderate (0.25)
CADD	33
Revel	Deleterious (0.97)
Varity	Deleterious (0.99)
SIFT	Deleterious (0)
MT	Deleterious (1)
ACMG criteria	PS3, PM2, PP3, PP5
Interpretation	Pathogenic

### 3.2 Functional Analysis Using Yeast Model System

**FIGURE 2:**
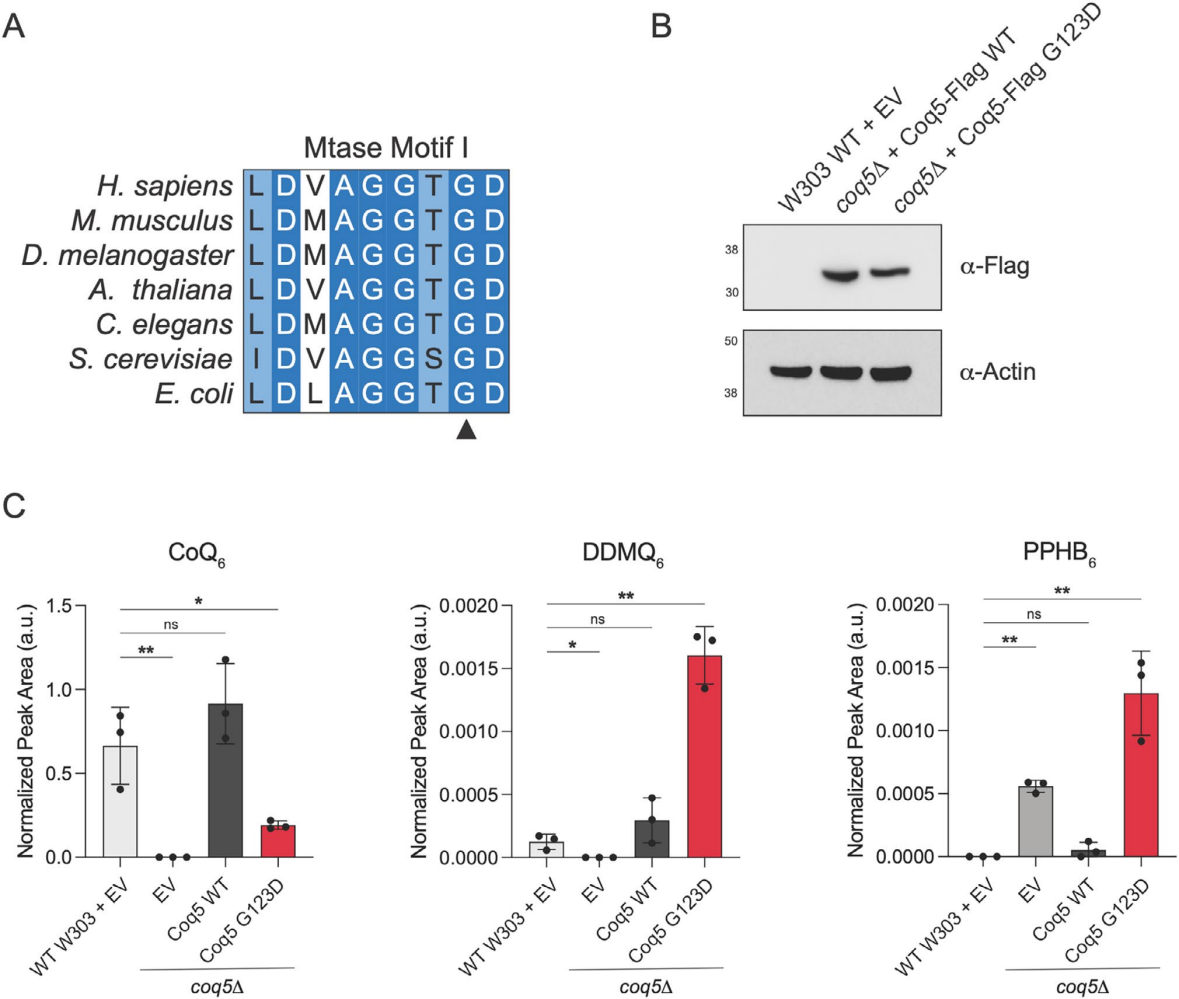
Yeast *COQ5* ^*G123D*^ does not support CoQ_6_ biosynthesis in *coq5*Δ. (A) Multiple sequence alignment of methyltransferase motif I of COQ5 homologs. Dark blue represents highly conserved residues, and light blue represents moderately conserved residues. The (▲) denotes human Gly118. (B) Western blotting of WT or *coq5*Δ yeast expressing EV or the indicated Coq5‐Flag construct under the control of the constitutive GPD promoter. (C) Relative total CoQ_6_, DDMQ_6_, and PPHB_6_ abundance of indicated Coq5‐Flag construct under the control of the constitutive GPD promoter. MS2‐based peak area is normalized to the CoQ_8_ internal standard. *n* = 3 biologically independent samples, two‐sided Student's *t*‐test, *p < 0.05, **p < 0.01.

Since the variant p.Gly118Asp does not cause splicing aberration, the effect of the variant on the protein had yet to be elucidated. We employed a Baker's yeast model to evaluate the functional impact of this variant using a homologous complementation approach. The residue p.Gly118 in human COQ5 corresponds to the conserved residue p.Gly123 in yeast *COQ5* within the first methyltransferase motif (Figure 2A). Direct mutagenesis to the residue p.Gly123 was then performed to generate *coq5* ^*G123D*^, which is equivalent to human p.Gly118Asp.

Targeted lipidomics was then performed to measure CoQ_6_ levels and intermediates in the CoQ_6_ biosynthetic pathway in *coq5*Δ harboring empty vector, wild‐type yeast *COQ5‐FLAG* (WT), or *COQ5* ^*G123D*^ *‐FLAG*. Western blotting was performed to validate the expression of Coq5‐Flag WT and G123D; Coq5‐Flag G123D was expressed at a slightly lower level compared to WT, indicating that the point mutation could have a small impact on protein stability in addition to catalytic activity (Figure 2B). The *coq5*Δ strain expressing an empty vector (EV) revealed undetectable CoQ_6_, while the *coq5*Δ strain with WT was able to fully rescue CoQ_6_ biosynthesis. Interestingly, the *coq5* ^*G123D*^ mutation was unable to rescue CoQ_6_ levels (Figure 2C). Correspondingly, the *coq5* ^*G123D*^ mutant exhibited a significant increase in the intermediate DDMQ_6_, the substrate of Coq5, as well as the earlier intermediate PPHB_6_, which commonly accumulates when the CoQ pathway is dysfunctional. The result indicates the blockade at the Coq5 reaction step, reflecting a loss‐of‐function impact of the variant p.Gly118Asp on the COQ5 enzyme function.

## 4 Discussion

Prior to this study, seven individuals from five families have been reported with *COQ5*‐PCD [9, 10, 11, 12]. Previous reports described patients with a range of clinical manifestations, including retinitis pigmentosa, with or without associated systemic involvement, as well as primarily neurological symptoms, varying in severity. Only one patient has been documented with severe multi‐systemic involvement. Here, we present two additional cases, both exhibiting severe multi‐systemic disease, thereby increasing the total number of reported patients to nine, derived from six families. Among those, most patients presented with developmental delay and intellectual disability (7/9, 77.8%). Seizure and ataxia are also common (62.5% (5/8) and 60% (3/5), respectively; Table 2). Cerebellar atrophy is frequently associated with *COQ5‐*PCD; however, this was not found in our patients, but instead had other brain MRI abnormalities classically associated with primary mitochondrial disorders, such as abnormal signal hyperintensity in basal ganglia and thalami. Most notably, brain imaging in our patients revealed multiple strokes, which, although associated with other mitochondrial disorders, have not been reported in patients with *COQ5*‐PCD [24].

**TABLE 2:** Clinical, biochemical and molecular characteristics of known patients with *COQ5*‐related primary CoQ_10_ deficiency.

Patient	Najmabadi et al. [12]	Malicdan et al. [9]			Dawidziuk et al. [11]	Jurkute [10]		Present study	
	M144	III.4	III.3	III.6		Family 11	Family 12	Patient 1	Patient 2
Variant	c.352G>A (p.Gly118Ser)	c.575‐1761_*1489dup (p.?)	c.575‐1761_*1489dup (p.?)	c.575‐1761_*1489dup (p.?)	c.352G>A (p.Gly118Ser) c.681 + 1G>A (p.?)	c.933delC (p.Tyr311*) c.682–7>G	c.367C>T (p.Arg123Trp) c.682–7>G	c.177_178del (p.Ser60Glyfs13) c.353G>A (p.Gly118Asp)	c.177_178del (p.Ser60Glyfs13) c.353G>A (p.Gly118Asp)
Zygosity	Homozygous	Homozygous	Homozygous	Homozygous	Compound heterozygous	Compound heterozygous	Compound heterozygous	Compound heterozygous	Compound heterozygous
Ethnicity	N/A	Iraqi‐Jewish	Iraqi‐Jewish	Iraqi‐Jewish	Polish	N/A	N/A	White	White
Gender	N/A	Female	Female	Female	Female	Male	Female	Female	Male
Consanguinity	Yes	No	No	No	No	N/A	N/A	No	No
Age at onset	N/A	N/A	N/A	N/A	5 m	N/A	N/A	6 m	3 m
Age at examination	N/A	N/A	N/A	N/A	10 y	56 y	38 y	27 months	7 years
Neurologic									
Developmental delay (mild/moderate/severe)	Severe	Moderate	None	Mild	Severe	—	—	Severe	Severe
Intellectual disability	N/A	N/A	Borderline	Mild	Yes	—	—	N/A	N/A
Microcephaly	N/A	N/A	N/A	N/A	Yes	—	—	Yes	Yes
Spasticity	N/A	Yes (LL)	N/A	N/A	Yes (LL)	—	—	Yes	Yes
Stroke‐like	N/A	—	—	—	—	—	—	Yes	Yes
Ataxia	N/A	Yes	Yes	Yes	N/A	—	—	N/A	N/A
Seizure	N/A	Yes	Yes	Yes	No	—	—	Yes	Yes
Dysarthria	N/A	Yes	Yes	Yes	Pronounce a few simple syllables	—	—	Nonverbal	Nonverbal
Ophthalmologic (optic nerve atrophy, retinopathy, cataract, strabismus, ptosis, ie)	N/A	Horizontal nystagmus, slow saccades with saccadic pursuit and apraxic gaze	Oculomotor apraxia, nystagmus	Horizontal nystagmus, hypometric saccades.	Normal	RP	RP, nystagmus	RP, optic atrophy	Esotropia
Other clinical manifestations	N/A	Short stature, impulsivity, attention deficit, oppositional characteristics	N/A	N/A	Short stature, impulsivity, attention deficit	Hypertension	Weakness, Under developed fertile function	Feeding difficulties	Feeding difficulties
Brain MRI	N/A	Mild non‐progressive cerebellar atrophy	Mild cerebellar atrophy	N/A	Progressive cerebellar atrophy	N/A	N/A	Abnormal signal intensity in bilateral medial thalami and dorsal midbrain, recurrent strokes	Encephalomalacia, hyperintensities in the bilateral thalami and brainstem, multiple strokes of varying age
Respiratory chain enzymes	N/A	Decreased Complex II + III activity	N/A	N/A	Normal	N/A	N/A	N/A	N/A
CoQ10 level (normal)		Leukocyte: 65 pmol/ug protein (119.86 ± 24.23); Muscle 21.53 μg/g tissue (26.63–48.11)	Leukocyte: 78 pmol/μg protein (119.86 ± 24.23)	Leukocyte: 72 pmol/μg protein (119.86 ± 24.23)	Plasma: 0.6 mg/L (> 0.67 mg/L)	Plasma: 582.28 nmol/L (227–1432)	None	Leukocyte: 38 pmol/mg protein (66–183 pmol)	Leukocyte: 39 pmol/mg protein (66–183 pmol)
Treatment	N/A	CoQ10 15 mg/kg/day	CoQ10 15 mg/kg/day	CoQ10 15 mg/kg/day	QuinoMit Q10 Fluid at 26 mg every second day	None	None	CoQ10 15 mg/kg/day	None

Abbreviations: LL, lower legs; N/A, Data not available; RP, retinopathy.

Ophthalmologic involvement is common among previously reported patients (6/7, 86%). Eye movement disorders were reported in three patients [9], while RP was a major presentation in two patients [10]. However, optic atrophy has not been previously reported. Thus, our study further supports the link between *COQ5* variants and RP, expanding the ophthalmologic phenotype in *COQ5*‐PCD. Unlike other primary CoQ_10_ deficiencies, cardiac and renal involvement have not been reported in *COQ5*‐PCD.

Though our patients had low CoQ_10_ levels in blood, these are not consistently low in *COQ5*‐PCD, with 85% of the patients (6/7) having decreased CoQ_10_ levels in plasma, leukocyte, or muscle specimens [9, 11]. This variability may be partly due to differences in methodology or tissue sampling, in addition to phenotypic heterogeneity. This highlights the crucial role genetic testing plays in diagnostic confirmation.

Malicdan et al. described a complex intronic variant in the homozygous state that led to reduced COQ5 mRNA and protein expression, supporting a loss‐of‐function mechanism in the pathogenesis of COQ5‐PCD [9]. Subsequently, one nonsense (p.Tyr311Ter) and two canonical splicing variants (c.682–7>G and c.681 + 1G>A) have been reported. Additionally, two missense variants (p.Arg123Trp, and p.Gly118Ser) have been identified; however, their clinical significance remains uncertain. This is partly due to the limited number of missense variants reported to date and the limited predictive accuracy of *in silico* tools. Furthermore, no functional studies had been conducted to establish the deleterious effects of these missense variants, which limits the strength of the variant‐disease association.

In our current report, our patients presented with the p.Gly118Asp variant in *trans* with a frameshift variant. The missense variant was located in an exon‐intron boundary, but RNA analysis revealed that the variant does not affect splicing. In order to study the potentially disruptive effect of this variant on the biochemical activity of COQ5, we employed a yeast system. Given its facultative anaerobe nature and its highly conserved mitochondrial biology, *S. cerevisiae* has been used in studies of mitochondrial disorders and proven effective for evaluating the impact of variants on CoQ biosynthesis [25, 26]. In this study, we measured yeast CoQ_6_, an equivalent to human CoQ_10_, and intermediates in the CoQ_6_ biosynthetic pathway. This revealed that yeast harboring a variant equivalent to human p.Gly118Asp had decreased CoQ_6_ levels accompanied by an accumulation of the COQ5 yeast‐equivalent substrate, DDMQ_6_. These findings support the loss‐of‐function effect of the p.Gly118Asp variant and further emphasize the use of yeast as a model system for *COQ5*‐PCD as well as other primary CoQ_10_ deficiencies.

Interestingly, two missense variants affecting the same amino acid residue, p.Gly118Ser and p.Gly118Asp, have been identified in *COQ5*‐PCD. The patient homozygous for p.Gly118Ser exhibited profound developmental delay, although information regarding systemic involvement was unavailable [12]. A patient with compound heterozygosity for p.Gly118Ser and a splice variant presented with severe multisystemic disease [11]. These observations suggest that glycine at position 118 plays a crucial role in COQ5 function, and variants affecting this residue may be associated with more severe multisystemic COQ5‐PCD. The Gly118 residue is highly conserved and resides within the DVAGGSG segment, which is essential for the binding of S‐adenosylmethionine (SAM), a key methyl donor [27]. Disruption of this segment may impair SAM binding, leading to a significant loss of enzyme function. These findings may suggest a genotype–phenotype correlation in *COQ5*‐PCD.

CoQ_10_ supplementation is one of the treatment strategies in primary CoQ_10_ deficiencies; however, patients' responses could be variable [6]. Patients with *COQ5*‐PCD with ataxia as a predominant symptom have shown subjective improvement in ataxia following CoQ_10_ supplementation [9]. Therefore, we supplemented patient 1 with 15 mg/kg/day of CoQ_10_. The parents reported subjective improvement in her social interaction. There was also a suggestion of reduced stroke risk in that surveillance brain MRIs did not show new strokes while the patient was supplemented. It is possible that CoQ_10_ supplementation may be beneficial among patients with *COQ5*‐PCD, especially among the patients with reduced CoQ_10_ levels. However, given some of the reported patients maintained normal CoQ_10_ levels, it is likely that decreased CoQ_10_ is not the only underlying pathophysiology of *COQ5*‐PCD, as well as other primary CoQ_10_ deficiencies [10]. Therefore, supplementation of CoQ_10_ may provide benefit only in certain patients. Larger cohorts and a more complete understanding of the disease are needed to provide robust evidence for the benefit of CoQ_10_ supplementation [3].

Here, we described a pair of siblings with *COQ5*‐PCD and demonstrated the use of a yeast system as a model to study *COQ5*‐PCD. We also highlighted intrafamilial variability and demonstrated that recurrent stroke and optic atrophy are possible manifestations of *COQ5*‐PCD. Given the variable degree of previously reported patients ranging from isolated RP to severe multisystemic disorders, larger cohorts of patients are needed to establish neurologic and extra‐neurologic phenotypes, as well as genotype–phenotype correlation. This study expanded the genotypic and phenotypic spectra of the disease.

## Author Contributions

P.W. and J.L.G. designed and conceptualized the study. P.W., S.T., J.A.H., and J.L.G. performed clinical analysis of the patients. P.W., J.L.G., and D.J.W. performed variant analysis. D.J.W. performed RNA analysis. R.M.G. and D.J.P. performed functional study in yeast model system. P.W. drafted the manuscript. All authors were involved with revising the manuscript. P.W. and J.L.G. obtained consents. P.W., J.L.G., and D.J.P. supervised the study.

## Ethics Statement

The guardian of the patient signed a consent form for publication approved by Washington University IRB (Media Authorization for the Use and Disclosure of Protected Health Information).

## Consent

Consent was obtained from the patient's family for publication of this report.

## Conflicts of Interest

The authors declare no conflicts of interest.

## Supporting information


**Figure S1.** RNA studies. (A) Splicing prediction in Alamut Software. Variant allele shows a mild decrease in 3′ splice site strength. (B) Primers used to amplify exons 1–4 of COQ5 cDNA. (C) Agarose gel electrophoresis of exons 1–4 of COQ5 cDNA PCR from proband. Lane 1: DNA ladder, lanes 2–3: Proband, lane 4: mom, lane 5: dad. (D) Deep next generation sequencing of cDNA PCR product. No alternative splicing was seen in 2 proband samples nor a control sample, despite sequencing to depths of > 1500 reads. 82% of reads contained the G118D mutant allele, while only 18% of reads contained the GT deletion allele, confirming degradation of the GT deletion allele, likely due to nonsense mediated decay.

## Data Availability

The authors have nothing to report.
