# Supplementary material for: Severe Neurological Presentation in Siblings With COQ5 ‐Related Primary Coenzyme Q10 Deficiency: Expanding Clinical and Molecular Spectrum
Source: JIMD Rep. 2025 Nov 5;66(6):e70038. doi: 10.1002/jmd2.70038 (PMC12588166; doi:10.1002/jmd2.70038)
Supplement: Supplementary file 1 — Figure S1. RNA studies. (A) Splicing prediction in Alamut Software. Variant allele shows a mild decrease in 3′ splice site strength. (B) Primers used to amplify exons 1–4 of COQ5 cDNA. (C) Agarose gel electrophoresis of exons 1–4 of COQ5 cDNA PCR from proband. Lane 1: DNA ladder, lanes 2–3: Proband, lane 4: mom, lane 5: dad. (D) Deep next generation sequencing of cDNA PCR product. No alternative splicing was seen in 2 proband samples nor a control sample, despite sequencing to depths of > 1500 reads. 82% of reads contained the G118D mutant allele, while only 18% of reads contained the GT deletion allele, confirming degradation of the GT deletion allele, likely due to nonsense mediated decay. [file JMD2-66-e70038-s001.pptx]

## Slide 1
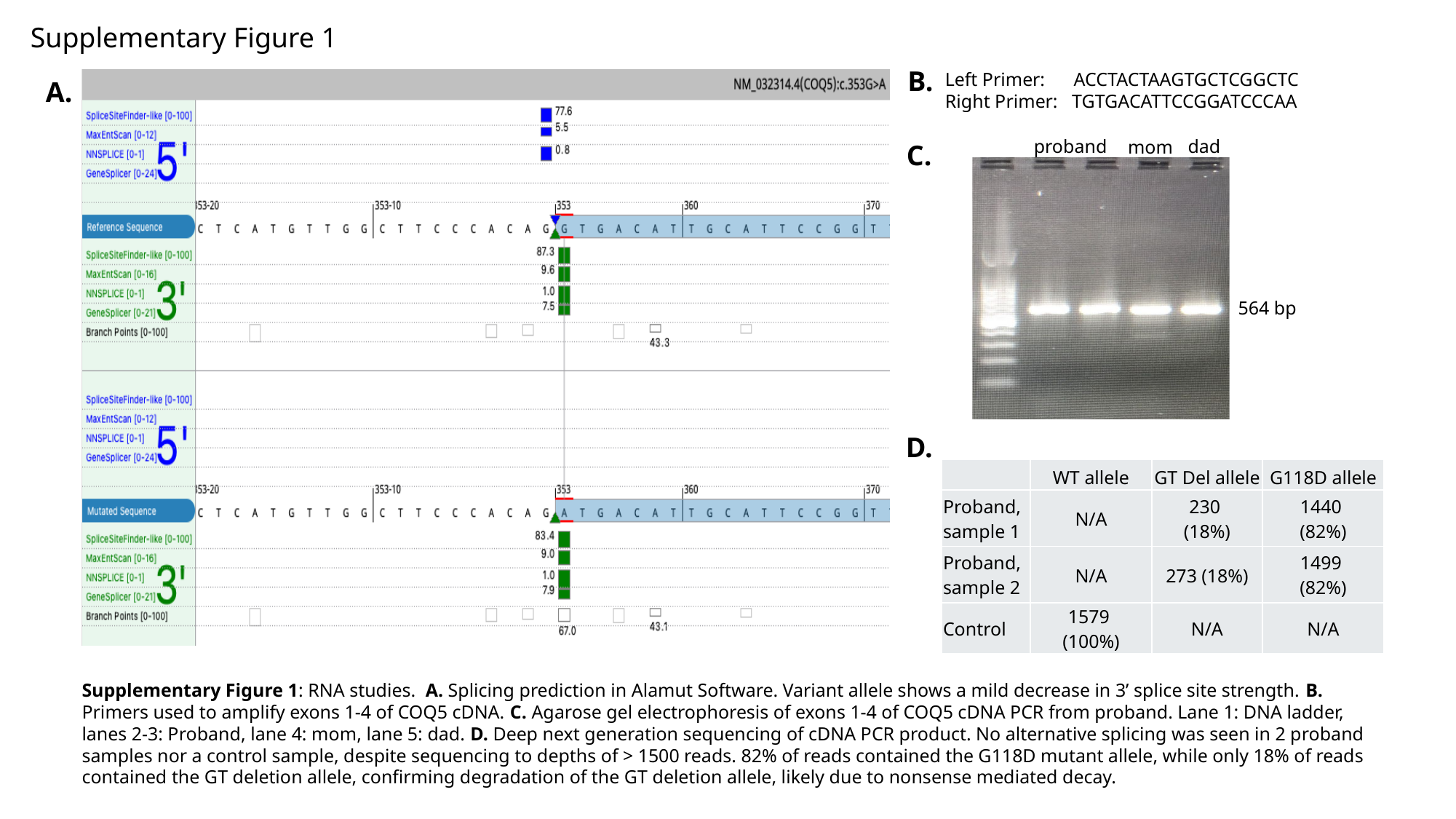

Supplementary Figure 1
B.
Left Primer: ACCTACTAAGTGCTCGGCTC
Right Primer: TGTGACATTCCGGATCCCAA
A.
proband
dad
mom
C.
564 bp
D.
| | WT allele | GT Del allele | G118D allele |
| --- | --- | --- | --- |
| Proband, sample 1 | N/A | 230 (18%) | 1440 (82%) |
| Proband, sample 2 | N/A | 273 (18%) | 1499 (82%) |
| Control | 1579 (100%) | N/A | N/A |
Supplementary Figure 1: RNA studies. A. Splicing prediction in Alamut Software. Variant allele shows a mild decrease in 3’ splice site strength. B. Primers used to amplify exons 1-4 of COQ5 cDNA. C. Agarose gel electrophoresis of exons 1-4 of COQ5 cDNA PCR from proband. Lane 1: DNA ladder, lanes 2-3: Proband, lane 4: mom, lane 5: dad. D. Deep next generation sequencing of cDNA PCR product. No alternative splicing was seen in 2 proband samples nor a control sample, despite sequencing to depths of > 1500 reads. 82% of reads contained the G118D mutant allele, while only 18% of reads contained the GT deletion allele, confirming degradation of the GT deletion allele, likely due to nonsense mediated decay.
